# Prognostic Nomogram for Childhood Acute Lymphoblastic Leukemia: A Comprehensive Analysis of 673 Patients

**DOI:** 10.3389/fonc.2020.01673

**Published:** 2020-09-10

**Authors:** Rui Mao, Shaoxuan Hu, Yuanchuan Zhang, Feng Du, Yu Zhang, Yanjun Liu, Tongtong Zhang

**Affiliations:** ^1^The Affiliated Hospital of Southwest Jiaotong University, Chengdu, China; ^2^Department of Hematology, Peking University Cancer Hospital and Institute, Beijing, China; ^3^The Center of Gastrointestinal and Minimally Invasive Surgery, The Third People’s Hospital of Chengdu, Chengdu, China; ^4^Key Laboratory of Carcinogenesis and Translational Research, Ministry of Education, Peking University Cancer Hospital and Institute, Beijing, China; ^5^State Key Laboratory of Molecular Oncology, National Cancer Center, National Clinical Research Center for Cancer, Cancer Hospital, Beijing, China; ^6^Medical Research Center, The Third People’s Hospital of Chengdu, The Affiliated Hospital of Southwest Jiaotong University, The Second Chengdu Hospital Affiliated to Chongqing Medical University, Chengdu, China

**Keywords:** childhood acute lymphoblastic leukemia, prognosis, nomogram, therapeutically applicable research to generate effective treatment database, genetic predisposition

## Abstract

**Objective:**

Despite that the survival rate in childhood acute lymphoblastic leukemia (cALL) is excellent, subsets of high-risk patients with cALL still have high relapse rates, and the cure rate is well below that for which we should aim. The present study aims to construct a prognostic nomogram to better inform clinical practitioners and improve risk stratification for clinical trials.

**Methods:**

The developed nomogram was based on the therapeutically applicable research to generate effective treatment (TARGET) database. With this database, we obtained 673 cALL patients with complete clinical information. We identified and integrated significant prognostic factors to build the nomogram model by univariate and multivariate Cox analysis. The predictive accuracy and discriminative ability of the nomogram were determined by the concordance index (C-index), calibration curve, and area under the receiver operating characteristic (ROC) curve (AUC) of ROC analysis. Internal validations were assessed by the bootstrapping validation.

**Results:**

In the multivariate analysis of the primary cohort, the independent factors for survival were ETV6 RUNX1 fusion status, karyotype, minimal residual disease (MRD) at day 29, and DNA index, which were all integrated into the nomogram. The calibration curve for the probability of survival showed good agreement between the prediction by the nomogram and the actual observation. The C-index of the nomogram for predicting survival was 0.754 (95% CI, 0.715–0.793), and the AUCs for 3-, 5-, and 7-year survival were 0.775, 0.776, and 0.772, respectively.

**Conclusion:**

We comprehensively evaluated the risk of clinical factors associated with prognosis and carried out risk stratification. The nomogram proposed in this study objectively and accurately predicted the prognosis of children with ALL.

## Introduction

Acute lymphoblastic leukemia (ALL) is the most common cancer in children and represents approximately one quarter of all cancers among persons younger than 15 years (1). The cure rate of ALL is increasing (2), but approximately 15–25% of patients will relapse after recovery, which is the leading cause of death in childhood acute lymphoblastic leukemia (cALL) patients (3, 4). Therefore, it is particularly important to determine the factors that affect the prognosis of cALL.

Many reported factors are related to the prognosis of cALL, such as ETV6 RUNX1 fusion status (5), mixed-lineage leukemia (MLL) gene rearrangement (6), TCF3 PBX1 fusion status (7), BCR-ABL1 fusion status (8), trisomy of leukemic cell chromosomes 4 and 10 (TRISOMY 4 10 status), or all usual combinations of trisomies (chromosomes 4, 10, 17, and 18) (9, 10), minimal residual disease (MRD) status in bone marrow of day 29 (11), the percent of blasts in bone marrow aspirate at day 29 of induction therapy (BMA blasts day 29) (12), cALL with Down syndrome (DS) (5), hypodiploid (DNA index <0.8) (13), and white blood cell (WBC) count at diagnosis (14–16). However, no study has used the whole series of factors as a prognostic model to predict the prognosis of cALL.

Therapeutically Applicable Research to Generate Effective Treatment (TARGET) is a dynamically updated database of the National Cancer Institute (NCI)’s Office of Cancer Genomics (OCG), whose mission is to advance the molecular understanding of cancers with the goal of improving patient outcomes. cALL is one of the projects in the TARGET program, which includes phase I (B-ALL), phase II (B-ALL, T-ALL), and phase III (ALL). We used a complete sample of all relevant clinical features in phases 1 and 2, which included 1,842 ALL patients.

Currently, nomograms have been developed for the majority of cancer types. The use of nomograms has compared favorably to the traditional staging systems for many cancers (17–19), and thus, they have been proposed as an alternative or even as a new standard (20–22). To our knowledge, this study is the first attempt to establish a prognostic nomogram for cALL based on 1,842 cALL samples in the TARGET database.

## Materials and Methods

### Patients

**FIGURE 1:**
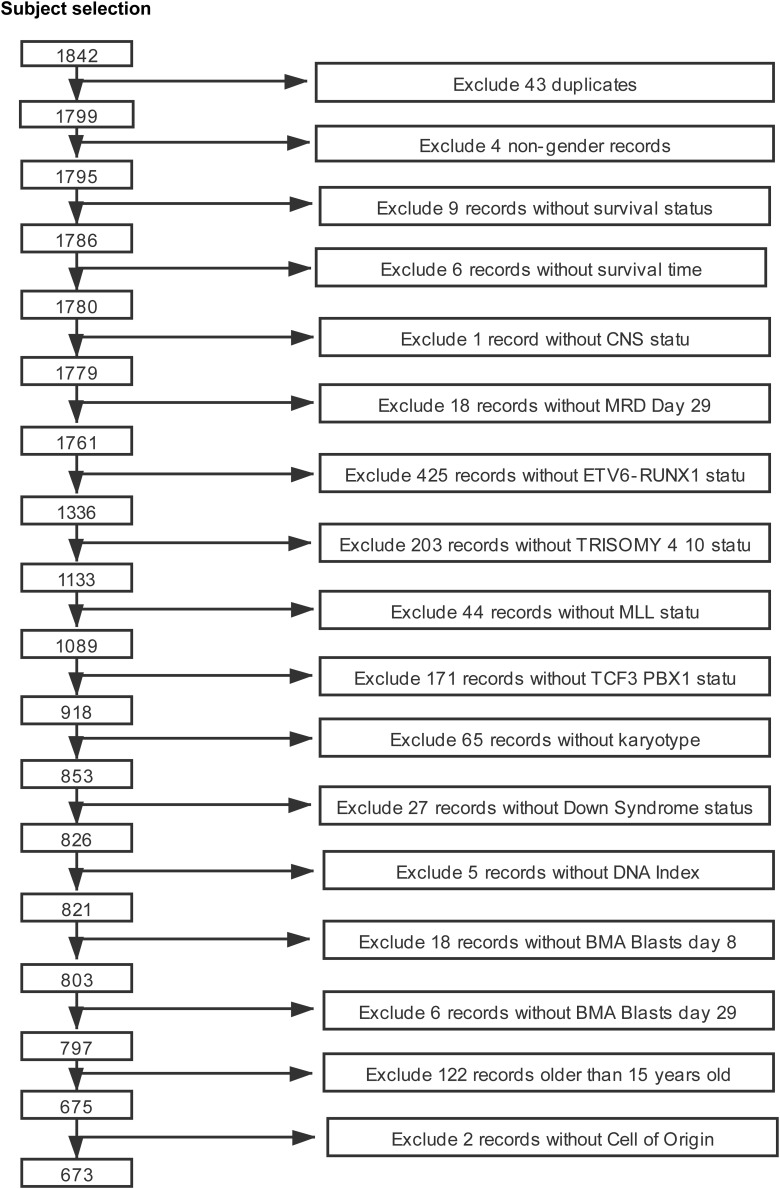
The screening process for the study subject.

Clinical parameters associated with childhood ALL patients up to June 10, 2019 were downloaded from the NCI TARGET database^1^. A total of 1,842 patients were included in the first and second phases of the data set. Of these subjects, patients with cALL who had information of main observation indicators were target subjects of this study, which include survival time, survival status, age at diagnosis, gender, ETV6 RUNX1 fusion status, TRISOMY 4 10 status, MLL status, TCF3 PBX1 fusion status, karyotype, BCR-ABL1 fusion status, central nervous system (CNS) status at diagnosis, BMA blasts day 8, BMA blasts day 29, cell of origin, Down syndrome status, MRD at day 29, and DNA index. The specific screening process is presented in Figure 1. The clinicopathological characteristics of patients in the cohorts are listed in Table 1. All source data are presented in Supplementary Table S1.

**TABLE 1:** The clinicopathological characteristics of patients in the cohort.

Characteristics	Target-ALL (*N* = 673)	
	Alive	Dead
Survival status, *n* (%)	553 (82.2)	120 (17.8)
**Age (years)**		
Median	5.5	
Range	1.0–15.0	
**Gender, *n* (%)**		
Male	293 (53.0)	66 (55.0)
Female	260 (47.0)	54 (45.0)
**ETV6 RUNX1 Fusion Status, *n* (%)**		
Positive	106 (19.2)	4 (5.0)
Negative	447 (80.8)	116 (95.0)
**CNS Status, *n* (%)**		
CNS1	452 (81.7)	89 (74.2)
CNS2	77 (13.9)	28 (23.3)
CNS3	24 (4.4)	3 (2.5)
**TRISOMY 4 10 Status, *n* (%)**		
Positive	92 (16.6)	4 (3.3)
Negative	461 (83.4)	116 (96.7)
**Karyotype, *n* (%)**		
No trisomies in 4, 10, 17, 18	367 (66.3)	108 (89.9)
4, 10, 17, 18 have only one trisomy	43 (7.8)	5 (4.2)
Double trisomies	28 (5.1)	2 (1.7)
Triple trisomies	115 (20.8)	5 (4.2)
**MLL Status, *n* (%)**		
Positive	23 (4.2)	9 (7.5)
Negative	530 (95.8)	111 (92.5)
**TCF3 PBX1 Status, *n* (%)**		
Positive	38 (6.9)	15 (12.5)
Negative	515 (93.1)	105 (87.5)
**WBC at Diagnosis (×10^∧^3/mcL)**		
Median	27	
Range	0.7–1306	
**BCR-ABL1 Status, *n* (%)**		
Negative	543 (98.2)	115 (95.8)
Positive	10 (1.8)	5 (4.2)
**BMA Blasts Day 8 (%)**		
Median	9	
Range	0–97	
**BMA Blasts Day 29 (%)**		
Median	0	
Range	0–50	
**MRD of 29 day (%)**		
Median	0	
Range	0–43	
**Cell of Origin, *n* (%)**		
B Cell ALL	500 (90.4)	115 (95.9)
T Cell ALL	53 (9.6)	5 (4.1)
**Down Syndrome, *n* (%)**		
Yes	5 (0.9)	5 (4.2)
No	548 (99.1)	115 (95.8)
**DNA Index**		
Median	1	
Range	0–1.922	

*WBC at Diagnosis, WBC count at diagnosis; CNS Status, CNS status at diagnosis.*

### Diagnosis

Age refers to the age at diagnosis. The WBC count at diagnosis was the absolute peripheral WBC count (in ×10^3^/mcL). CNS status at diagnosis was determined according to the status of CNS leukemia (CNSL) at the time of diagnosis. Diagnosis and typing were according to Pediatric Acute Lymphoblastic Leukemia, Version 2.2020, NCCN Clinical Practice Guidelines in Oncology (23). MRD status was determined by flow cytometry in bone marrow on day 29. BMA blasts day 29 and day 8 represent the percent of blasts in bone marrow aspirate at day 29 or day 8 of induction therapy. The presence or absence of ETV6 RUNX1 fusion status, MLL status, TCF3 PBX1 status, and BCR-ABL1 fusion status were detected by fluorescence *in situ* hybridization (FISH), PCR, or cytogenetics. The type of cell origin is determined by bone marrow examination. Karyotype analysis was determined by chromosomal banding technique to find abnormal chromosome number of leukemia cells and structural changes such as translocation, inversion, and deletion (23).

### Grouping of Clinical Characteristics

Age was divided into two groups based on the cutoff value of 10 years old (24, 25), and WBC count at diagnosis was divided into three groups: “<50,” “50 to 100,” and “≥100” (3, 26, 27) according to the WBC count (in ×10^3^/mcL). Next, CNS status at diagnosis was divided into three groups: CNS1, CNS2, and CNS3. MRD day 29, which means minimum residual disease status at day 29 of induction therapy, was divided into four groups: <0.01, 0.01–0.1%, 0.1–1%, and >1% (28, 29). BMA blasts day 29, which represents the percent of blasts in bone marrow aspirate at day 29 of induction therapy, was divided into two groups with a 5% cutoff. Besides, BMA blasts day 8 was divided into two groups according to whether it is >20%. The karyotypes are divided into four groups according to the trisomy of chromosomes 4, 10, 17, and 18, which include “no trisomies in 4, 10, 17, 18,” “4, 10, 17, 18 have only one trisomy,” “double trisomies (DT),” and “triple trisomies (TT).” Cell of origin contained two subtypes, which included B cell ALL and T cell ALL. Finally, the DNA index, whose number represents the ratio of the DNA content or chromosome number in a tumor sample compared to a normal diploid sample, was divided into two groups: ≤0.8 and >0.8. ETV6 RUNX1 fusion status, MLL status, TCF3 PBX1 status, and BCR-ABL1 fusion status were divided into positive and negative according to whether its corresponding fusion gene were positive or negative.

### Statistical Analyses

All data including demographic and disease characteristics were expressed as count (%). Statistical analysis was performed using the R software (Version 3.6.1)^2^.

The prognostic value of the 16 clinical characteristics was first calculated in the univariate Cox analysis; clinical features with a *P* < 0.1 in the multivariate Cox regression analysis were used to construct the nomogram via the “rms,” “survival,” and “foreign” packages of R (R version 3.6.1). Hazard ratio (HR) and 95% confidence interval (CI) were calculated. The performance of the nomogram was measured by the C-index and assessed by comparing the nomogram-predicted estimates versus the observed Kaplan–Meier estimates of survival probability (R package “rms”) (30). Based on the regression coefficients of the multivariate Cox regression analysis, a risk score composed of six clinical features in the nomogram was calculated, and the patients were divided into two groups by taking the corresponding median risk score as the cutoff point.

Kaplan–Meier curves and the log-rank test were used to compare the survival outcomes of the two groups with the R packages “survminer” and “survival” (R version 3.6.1). Receiver operating characteristic (ROC) curve analysis was employed to compare prediction concerning the accuracy and precision with the R package “survivalROC” (R version 3.6.1). Bootstrapping validation (1,000 bootstrap resamples) was used to calculate a relatively corrected C-index of the nomogram (31, 32). A *P* < 0.05 was considered significant.

## Results

### Clinicopathological Characteristics of the Patients

The cohort included 673 cALL patients with complete clinical information of main observation indicators. The median of age at diagnosis was 5.5 years (range, 1.0–15.0 years). Three-hundred fifty-nine (53.3%) patients in the cohort were male, while 314 patients were female. The median of survival time was 3,016 days (range, 28–5,598 days). Survival status showed 120 deaths and 553 survivals.

### Independent Prognostic Factors in the Primary Cohort

In the primary cohort, we performed a univariate Cox regression analysis for each clinical factor (Table 2) and screened factors with *P* < 0.1, which included age, WBC count at diagnosis, CNS status at diagnosis, ETV6 RUNX1 fusion status, TRISOMY 4 10 status, karyotype, TCF3 PBX1 fusion status, BCR-ABL1 fusion status, MRD day 29, BMA blasts day 8, Down syndrome, and DNA index. Then, these factors were analyzed in a multivariate Cox regression model (Table 3). The results show that the patient and disease variables significantly associated with survival in multivariable modeling included positive ETV6 RUNX1 fusion (HR = 0.293, 95% CI = 0.124–0.695, *P* = 0.005), triple trisomies (TT) (HR = 0.211, 95% CI = 0.049–0.907, *P* = 0.036), MRD day 29 with 0.01–0.1% (HR = 1.942, 95% CI = 1.113–3.330, *P* = 0.016), MRD day 29 with 0.1–1% (HR = 2.140, 95% CI = 1.262–3.628, *P* = 0.005), MRD day 29 > 1% (HR = 2.508, 95% CI = 1.345–4.675, *P* = 0.004), and DNA index ≤0.8 (HR = 3.617, 95% CI = 1.233–10.612, *P* = 0.019).

**TABLE 2:** The results of univariate Cox analysis.

Clinical characteristics	HR	Lower 95% *CI*	Upper 95% *CI*	*P*-value
**Age**				
≥10 years vs. <10 years	1.557	1.071	2.264	0.021*
**Gender**				
Male vs. Female	1.076	0.749	1.544	0.693
**ETV6 RUNX1 Fusion Status**				
Positive vs. Negative	0.233	0.102	0.529	0.0005***
**TRISOMY 4 10 Status**				
Positive vs. Negative	0.190	0.070	0.515	0.001**
**MLL Status**				
Positive vs. Negative	1.701	0.859	3.370	0.128
**WBC (×10^∧^3/mcL)**				
<50				
50 to 100	1.806	1.147	2.845	0.011*
>100	2.420	1.471	3.981	<0.001***
**Karyotype**				
No trisomies in 4, 10, 17, 18				
Only trisomies 4 or 10 or 17 or 18	0.464	0.189	1.137	0.093
Double trisomies (DT)	0.288	0.071	1.167	0.081
Triple trisomies (TT)	0.172	0.070	0.422	0.0001***
**CNS Status**				
CNS1				
CNS2	1.739	1.137	2.662	0.011*
CNS3	0.616	0.195	1.953	0.411
**TCF3 PBX1 Status**				
Positive vs. Negative	1.692	0.979	2.924	0.060
**BCR ABL1 Status**				
Positive vs. Negative	2.224	0.908	5.448	0.080
**MRD Day 29**				
<0.01%				
0.01 to 0.1%	1.973	1.176	3.309	0.01**
0.1 to 1%	2.890	1.809	4.615	8.88e-06***
>1%	3.959	2.276	6.884	1.10e-06***
**BMA Blasts Day 8**				
≥20% vs. <20%	1.760	1.228	2.521	0.002**
**BMA Blasts Day 29**				
≥5% vs. <5%	2.074	0.847	5.081	0.111
**Down Syndrome**				
Yes vs. No	3.144	1.283	7.704	0.012*
**Cell origin**				
T Cell ALL vs. B Cell ALL	0.483	0.197	1.184	0.112
**DNA Index**				
≤0.8 vs. >0.8	4.615	1.699	12.540	2.71e-03***

HR, hazard ratio; *CI*, confidence interval; WBC, WBC count at diagnosis; CNS Status, CNS status at diagnosis; * *P* < 0.05; ** *P* < 0.01; *** *P* < 0.001.

**TABLE 3:** The results of multivariate Cox analysis.

Clinical characteristics	HR	Lower 95% *CI*	Upper 95% *CI*	*P*-value
**Age**				
≥10 years vs. <10 years	1.164	0.784	1.726	0.452
**ETV6 RUNX1 Fusion Status**				
Positive vs. Negative	0.293	0.124	0.695	0.005**
**TRISOMY 4 10 Status**				
Positive vs. Negative	0.866	0.175	4.280	0.860
**WBC (×10^∧^3/mcL)**				
<50				
50 to 100	1.261	0.787	2.018	0.335
>100	1.539	0.912	2.627	0.114
**CNS Status**				
CNS1				
CNS2	1.465	0.937	2.293	0.094
CNS3	0.528	0.164	1.694	0.283
**TCF3 PBX1 Status**				
Positive vs. Negative	1.458	0.812	2.616	0.206
**BCR ABL1 Status**				
Positive vs. Negative	1.391	0.553	3.504	0.483
**MRD Day 29**				
<0.01%				
0.01 to 0.1%	1.942	1.133	3.330	0.016*
0.1 to 1%	2.140	1.262	3.628	0.005**
>1%	2.508	1.345	4.675	0.004**
**BMA Blasts Day 8**				
≥20% vs. <20%	1.354	0.890	2.058	0.157
**Down Syndrome**				
Yes vs. No	2.351	0.940	5.885	0.068
**DNA Index**				
≤0.8 vs. >0.8	3.617	1.233	10.612	0.019*
**Karyotype**				
No trisomies in 4, 10, 17, 18				
Only trisomies 4 or 10 or 17 or 18	0.515	0.207	1.278	0.152
Double trisomies (DT)	0.308	0.074	1.289	0.107
Triple trisomies (TT)	0.211	0.049	0.907	0.036*

HR, hazard ratio; *CI*, confidence interval; WBC, WBC count at diagnosis; CNS Status, CNS status at diagnosis; * *P* < 0.05; ** *P* < 0.01.

### Prognostic Nomogram for Overall Survival

**FIGURE 2:**
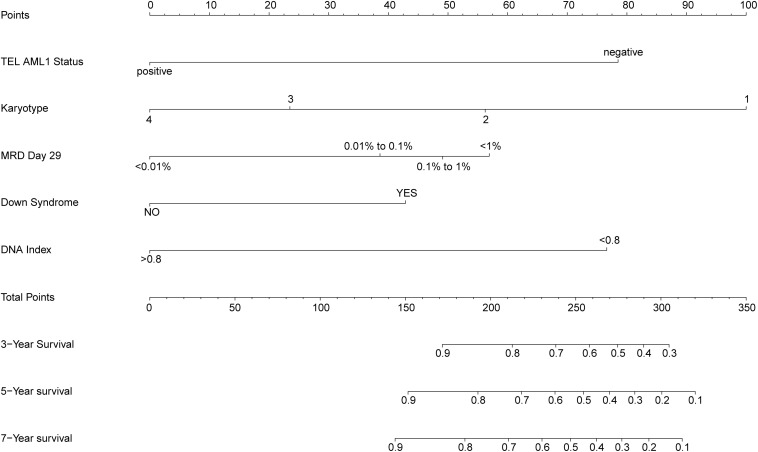
Prognostic nomogram for overall survival (OS). Childhood acute lymphoblastic leukemia survival nomogram. To use the nomogram, an individual patient’s value is located on each variable axis, and a line is drawn upward to determine the number of points received for each variable value. The sum of these numbers is located on the total points axis, and a line is drawn downward to the survival axes to determine the likelihood of 3-, 5-, and 7-year survival. CNS status, central nervous system status at diagnosis; MRD day 29, minimal residual disease status at day 29 of induction therapy; NA, no trisomies in 4, 10, 17, 18; T1, 4, 10, 17, and 18 have only one trisomy; DT, double trisomies; TT, triple trisomies.

**FIGURE 3:**
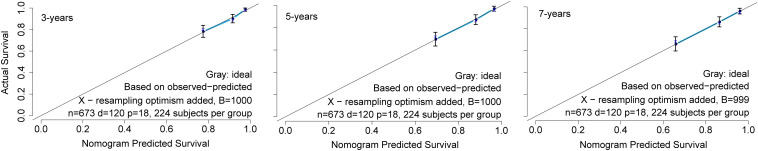
The calibration curve for nomogram. The calibration curve for predicting patient survival at 3, 5, and 7 years in the primary cohort. The nomogram-predicted probability of overall survival is plotted on the *x*-axis; the actual overall survival is plotted on the *y*-axis.

The prognostic nomogram that integrated all significant independent factors for overall survival (OS) in the primary cohort is shown in Figure 2. The nomogram demonstrated that the karyotype had the largest contribution to prognosis, followed by ETV6 RUNX1 fusion status, DNA index, and CNS status. BMA blast day 8 and MRD day 29 showed a moderate effect on survival rate. Each category within these variables was assigned a point on the top scale based on the coefficients from multivariate Cox regression. By summing all of the assigned points for the eight variables and drawing a vertical line between total points and survival probability axis, we were easily able to obtain the estimated probability of 3-, 5-, and 7-year survival. The C-index for OS prediction were 0.754 (95% CI, 0.715–0.793) and 0.731 for the primary cohorts and bootstrapping validation, respectively. The calibration plot for the probability of survival at 3, 5, and 7 years showed an optimal agreement between the predictions by the nomogram and the actual observations (Figure 3).

### ROC and Kaplan–Meier Curve Analyses

**FIGURE 4:**
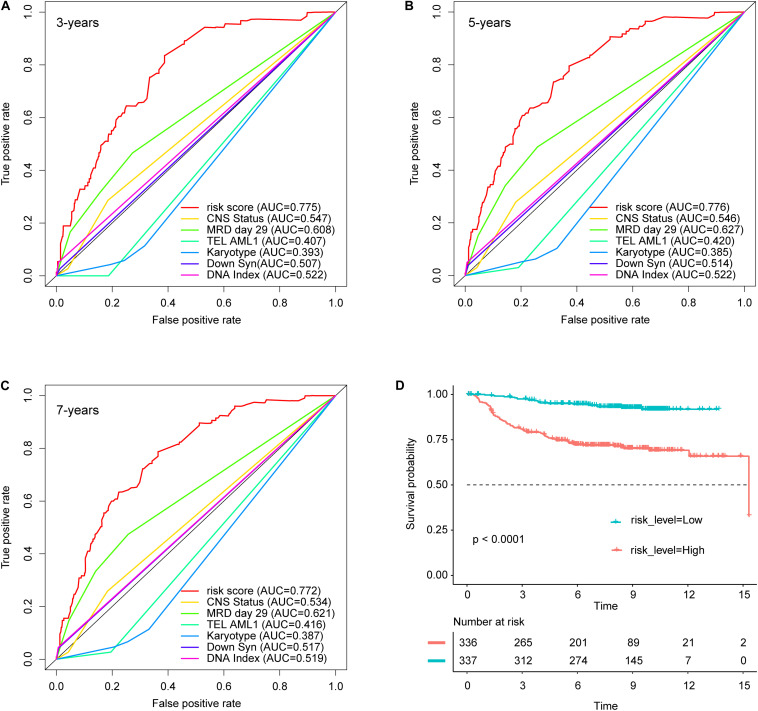
The results of receiver operating characteristic (ROC) and Kaplan–Meier curve analyses. Comparison of survival prediction with regard to specificity and sensitivity according to the risk score and clinical characteristics in the nomogram. The areas under the curve (AUCs) for **(A)** 3 years, **(B)** 5 years, and **(C)** 7 years are shown in the bottom right corner of each picture. **(D)** Kaplan–Meier curves comparing the survival outcomes of the two groups with the assistance of the log-rank test. CNS status, central nervous system status at diagnosis; MRD 29, minimal residual disease status at day 29.

We calculated the risk score, which was composed of six clinical features in the nomogram, based on the regression coefficients from the multivariate Cox regression analysis. We divided all patients in the training set into two groups, the high-risk score group (*n* = 336) and the low-risk score group (*n* = 337), by taking the corresponding median risk score as the cutoff point. Next, we compared survival predictions with regard to specificity and sensitivity according to the risk scores and clinical characteristics in the nomogram by ROC curve analysis. The results show that the areas under the curve (AUCs) of the risk score for 3-, 5-, and 7-year survival were 0.775, 0.776, and 0.772, respectively, which were higher than those of all of the clinical factors in the nomogram (Figures 4A–C). Patients with a high-risk score had a markedly worse OS than patients with a low-risk score (log-rank test *P* < 0.0001, Figure 4D).

### Survival Analysis for Subgroups According to Variable in Nomogram

**FIGURE 5:**
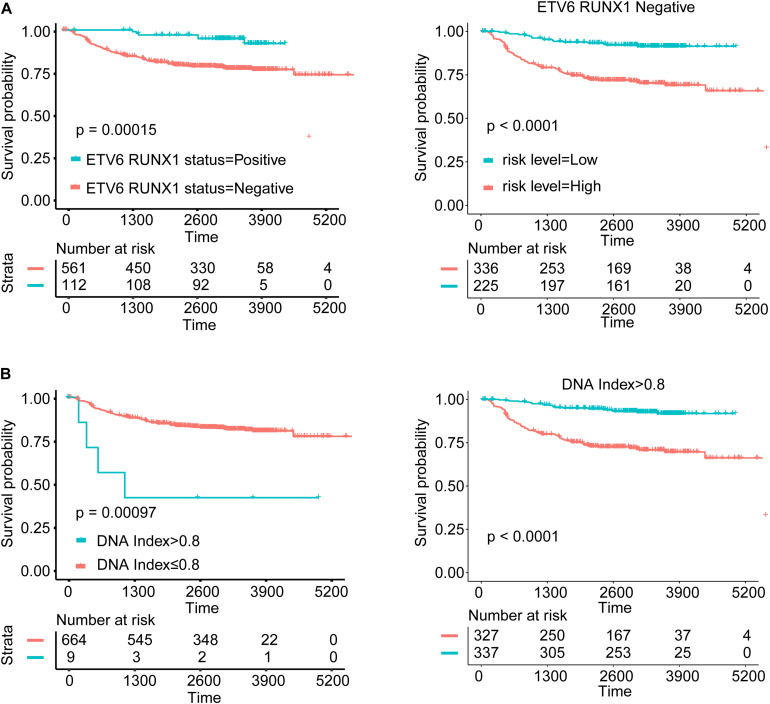
Survival analysis for subgroups according to variable in nomogram. **(A)** Kaplan–Meier curves of ETV6 RUNX1 fusion status and comparison of the survival outcomes between the high- and low-risk groups in different ETV6 RUNX1 fusion gene status. **(B)** Kaplan–Meier curves of DNA index status and comparison of the survival outcomes between the high- and low-risk groups in different DNA index status.

In general, subtype patients with positive ETV6 RUNX1 fusion gene showed better OS than patients with negative ETV6 RUNX1 fusion gene, and patients in the high-risk group had worse OS than those in the low-risk group in ETV6 RUNX1 fusion-negative segment, while in the ETV6 RUNX1 fusion gene-positive segment, all patients were in the low-risk group (Figure 5A). Besides, the Kaplan–Meier curves of OS suggested that hypodiploid patients had significantly worse OS than patients not. What is more, patients in the high-risk group had worse OS than those in the low-risk group in non-hypodiploid segment, and all patients of the hypodiploid segment were in the high-risk group (Figure 5B). These above results indicated that the risk scores we have obtained are quite accurate and predictive.

### Validated Nomogram in the Independent and Total Cohorts

To validate the robustness of our nomogram, we reviewed the data from the TARGET database again retrospectively, and the filtering process is presented in Supplementary Figure S1. We ended up with 299 valid cases of data as independent validation queues. Through a similar analysis process, we get supportive results. As Supplementary Figure S2 shows, in the validation cohort, the AUCs of the risk score for 3-, 5-, and 7-year survival were 0.683, 0.723, and 0.737, respectively, which were higher than those of all of the clinical factors in the nomogram (Supplementary Figure S2D). Patients with a high-risk score had a markedly worse OS than patients with a low-risk score (log-rank test *P* < 0.0001, Supplementary Figure S2A). The C-index of the nomogram for predicting survival was 0.703 (95% CI, 0.640–0.766). Besides, to further confirm the robustness of the model, we also validate the nomogram in the total cohort (*N* = 299 + 673 = 972). Patients with a high-risk score had a markedly worse OS than patients with a low-risk score (log-rank test *P* < 0.0001, Supplementary Figure S2C), and the AUCs of the risk score for 3-, 5-, and 7-year survival were 0.753, 0.783, and 0.773 (Supplementary Figure S2B), respectively. The C-index of the nomogram for predicting survival was 0.723 (95% CI, 0.684–0.762).

## Discussion

In the present study, we downloaded the data from TARGET database and screened patients with complete information of main observation indicators. Then, we identified independent prognostic factors by univariate and multivariate Cox regression analyses. Significant factors such as ETV6 RUNX1 fusion status, karyotype, MRD day 29, Down syndrome, and DNA index were applied to construct a prognostic nomogram. C-index, Kaplan–Meier analyses, ROC curves and AUC values show that the nomogram objectively and accurately predicted the prognosis of patients with cALL.

A large study showed that cases with trisomy of chromosomes 4, 10, 17, and 18 appear to have the most favorable outcome (10, 33–35). Besides, Harris et al. (9) found that, among patients with a DNA index >1.16, patients with trisomies of both chromosomes 4 and 10 had a 4-year EFS of 96.6% (*n* = 161, SE = 3.8%), whereas patients with neither or only one of these trisomies had a 4-year EFS of 70.4% (*n* = 73, SE = 11.5%). Convincingly, the Kaplan–Meier curves of OS suggested that patients with TT had significantly better OS than patients not in this study (HR = 0.211, *P* = 0.036).

The ETV6-RUNX1 fusion gene, which grew out of *t*(12; 21) (p13; q22) translocation, is the most common chromosome translocation abnormality among cALL. Rubnitz et al. (36, 37) found that the positive frequency of ETV6-RUNX1 in newly diagnosed and recurrent children was 25%, and the 5-year event-free survival (EFS) survival rate of positive children was more than 90%. A study with an average follow-up time of 8 years showed that the 5-year EFS of 244 ETV6-RUNX1-positive ALL children accounted (86 ± 2)%, while that of the ETV6-RUNX1-negative B-ALL children was (72 ± 2)% (38). Obviously, the fusion gene, ETV6-RUNX1, is associated with a favorable prognosis. Similarly, our results showed that the OS rate of ETV6-RUNX1-positive group was significantly higher than that of the group with negative ETV6-RUNX1 (HR = 0.293, *P* = 0.005).

Hypodiploid acute lymphoblastic leukemia (<44 chromosomes) comprises two subtypes with distinct transcriptional profiles and genetic alterations (33). Numerous studies have shown that hypodiploid (chromosome number ≤44) or DNA index <0.8 is a high-risk type for patients with cALL (13, 39, 40). Low-hypodiploid acute lymphoblastic leukemia has a very poor outcome (39). The frequency increases with age, from being extremely rare in children (<1%), to 5% in adolescents and young adults, and over 10% in adults (41). In our study, hypodiploid accounted for 1.52%, and OS was significantly inferior to non-hypodiploid (HR = 3.617, *P* = 0.019).

Current research suggests that MRD may be the main cause of relapse (42). MRD refers to the state of trace tumor cells that cannot be detected morphologically *in vivo* after the induction of remission or bone marrow transplantation in children with leukemia. It is considered to be a more objective and sensitive assessment of the specificity of the clinical treatment response and disease control. In the CCLG-ALL-2008 program of the Chinese Children’s Leukemia Collaborative Group, MRD has been used as an important indicator for risk stratification, and multiple studies have also shown that MRD can be used as an independent prognostic factor (42–44). In our study, KM analysis (log-rank test *P* < 0.0001, Supplementary Figure S4) and univariable COX regression analysis suggest that MRD has a great influence on survival of cALL patients. The presence of day 29 marrow MRD was associated with shorter OS in all risk groups; even patients with 0.01–0.1% day 29 MRD had poor outcome compared with patients negative for MRD patients (80.1 vs. 88.9% 5-year OS). Besides, multivariate COX regression analysis suggests that MRD is an independent risk factor in cALL patients, which was consistent with previous research results (45).

According to the proposed nomogram, we are able to estimate the 3, 5, and 7 years survival rate in patients with cALL, for example, a patient (TARGET-10-PARCVT) with negative TEL-AML1 (corresponds to 79 points), no trisomies in 4, 10, 17, and 18 (corresponds to 100 points), 0.8% of MRD day 29 (corresponds to 49 points), no Down Syndrome (corresponds to 0 points), and hypodiploid (corresponds to 77 points). The calculation according to the proposed nomogram is thus 79 + 100 + 49 + 77 = 305 points, predicting a 5-year survival rate of 17.5% postoperatively. In fact, she died with an OS of 237 days. Another example is that of a patient (TARGET-10-PAMEEK) with negative TEL-AML1 (corresponds to 79 points), no trisomies in 4, 10, 17, and 18 (corresponds to 100 points), 12.6% of MRD day 29 (corresponds to 57 points), no Down syndrome (corresponds to 0 points), and DNA index = 1 (corresponds to 0 points). The calculation according to the proposed nomogram is thus 79 + 100 + 57 = 236 points, predicting a 5-year survival rate of 60.1% postoperatively. In fact, she is still alive with an OS of 60 days, but he could be in danger due to the high value of MRD day 29. If the patient used the scoring system and control his MRD value <0.01%, his total score would be 179, with a 5-year survival rate of about 83.5%. There is a great significance for guiding clinical stratified treatment. Low-risk patients can appropriately reduce the intensity of treatment and do not need to do allogeneic bone marrow transplantation. Besides, high-risk patients need to consider more actively to do transplant and strengthen the consolidation of treatment after induction and remission.

Nomograms are a commonly used tool in oncology that can be used to calculate an individual probability by integrating diverse prognostic and determinant variables according to corresponding clinical characteristics (46, 47). At present, the study on the prognosis of cALL patients focuses on individual factors and lacks a prognostic model covering a comprehensive range of factors. In this study, a prognostic nomogram combining clinical factors was established. The clinical factors in the nomogram are not affected by researchers and can be easily obtained. Moreover, our nomogram had a better predictive accuracy than that of each factor alone. However, the limitation of this study is the lack of external validation. Due to the lack of the number of patients and the corresponding information, it is difficult to obtain relevant resources in public databases or disease centers. Multicenter prospective cohort study may predict patient’s prognosis more accurately.

## Conclusion

In conclusion, we comprehensively evaluated the risk of clinical factors associated with prognosis and carried out risk stratification. The nomogram proposed in this study objectively and accurately predicted the prognosis of children with ALL. This nomogram may be a useful tool that can help clinicians develop personalized treatment plans, thereby effectively improving the survival rate of cALL patients.

## Data Availability Statement

All datasets presented in this study are included in the article/Supplementary Material.

## Ethics Statement

As the data (TARGET datasets) are publicly available, no ethical approval was required.

## Author Contributions

RM, YL, and TZ conceived the project and designed the experiments. RM and SH wrote the manuscript. FD, YCZ, and YZ carried out the statistical analysis. YL, RM, and TZ contributed to manuscript revision. All authors provided suggestions during manuscript preparation and read the final version.

## Conflict of Interest

The authors declare that the research was conducted in the absence of any commercial or financial relationships that could be construed as a potential conflict of interest.

## Footnotes

1 https://ocg.cancer.gov/

2 https://www.R-project.org

## Abbreviations

ALL: acute lymphoblastic leukemia
AUC: area under the curve
cALL: childhood acute lymphoblastic leukemia
CI: confidence interval
CNS: central nervous system
DCA: decision curve analysis
HR: hazard ratio
MRD: minimal residual disease
NCI: National Cancer Institute
OCG: Office of Cancer Genomics
ROC: receiver operating characteristic
TARGET: Therapeutically Applicable Research to Generate Effective Treatment
WBC: white blood cell.
