# Supplementary material for: Prognostic Nomogram for Childhood Acute Lymphoblastic Leukemia: A Comprehensive Analysis of 673 Patients
Source: Front Oncol. 2020 Sep 10;10:1673. doi: 10.3389/fonc.2020.01673 (PMC7511595; doi:10.3389/fonc.2020.01673)
Supplement: Supplementary file 2 [file Data_Sheet_2.DOCX]

**Supplementary Figures**





**Supplementary Figure 1. The filtering process.**


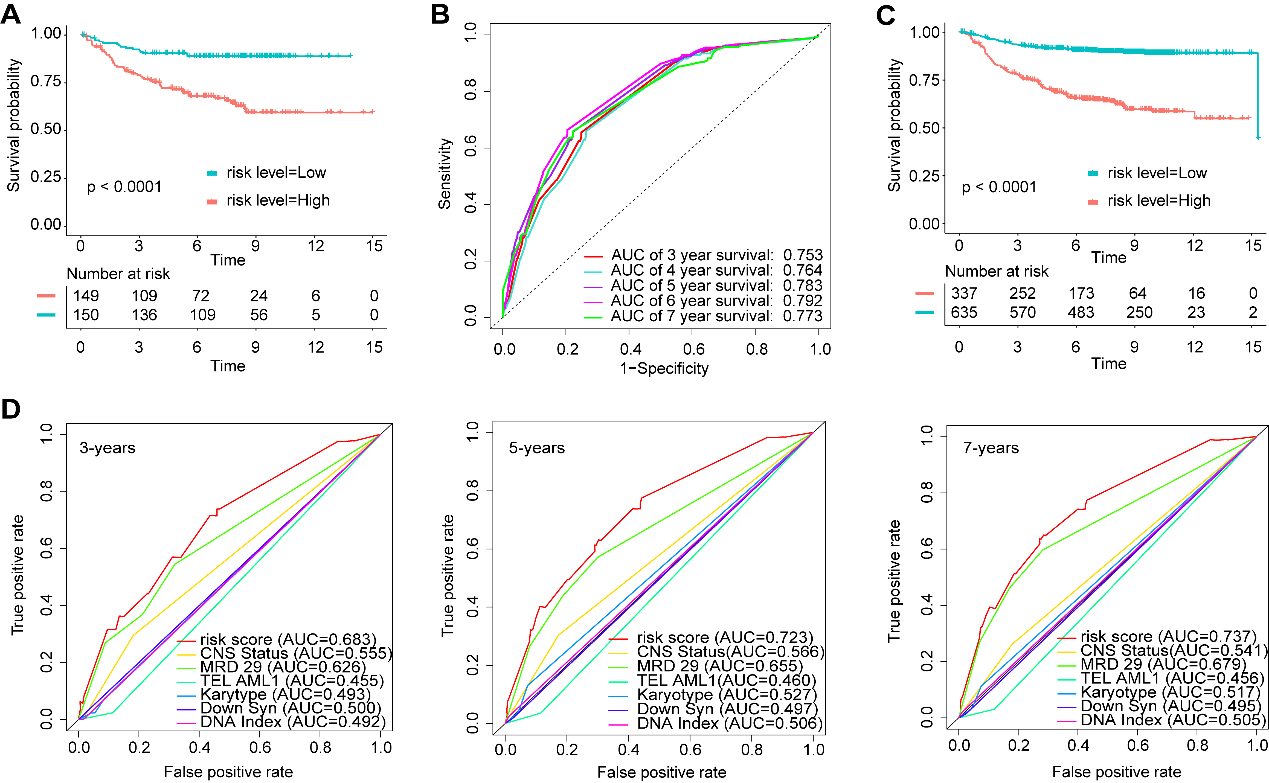


**Supplementary Figure 2. ROC, calibration, and Kaplan-Meier curve analyses for the nomogram in the validation and total cohort.** (**A**) Kaplan‐Meier curves comparing the survival outcomes of the two groups with the assistance of the log‐rank test in the validation cohort. The AUCs for 3, 4, 5, 6, and 7 years (**B**) of the total cohort are shown in the bottom right corner of the picture. (**C**) Kaplan‐Meier curves comparing the survival outcomes of the two groups with the assistance of the log‐rank test in the total cohort. (**D**) Comparison of survival prediction with regard to specificity and sensitivity according to the risk score and clinical characteristics in the validation cohort.


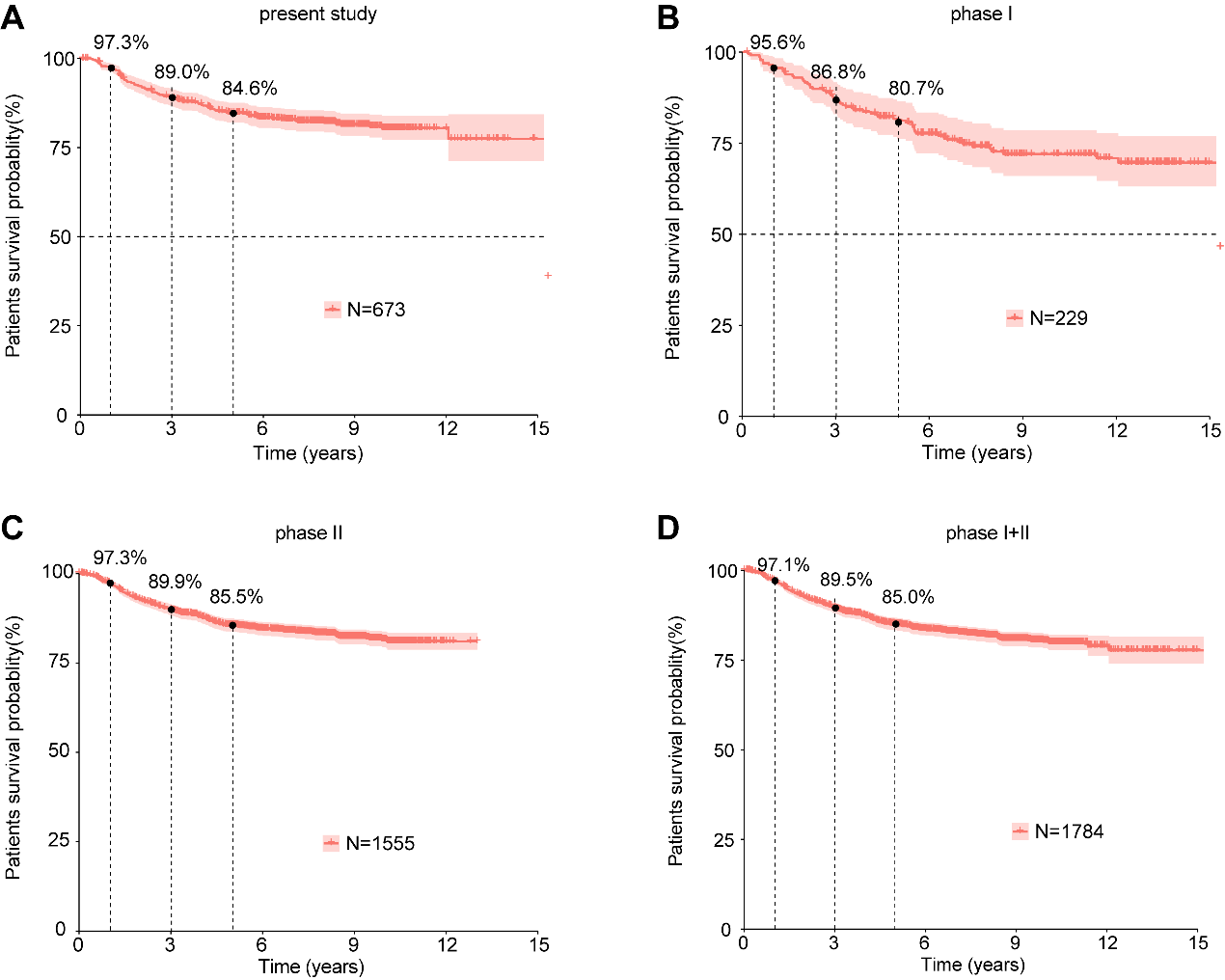


**Supplementary Figure 3.** 1,3,5-year survival rate for cALL patients in different queues.


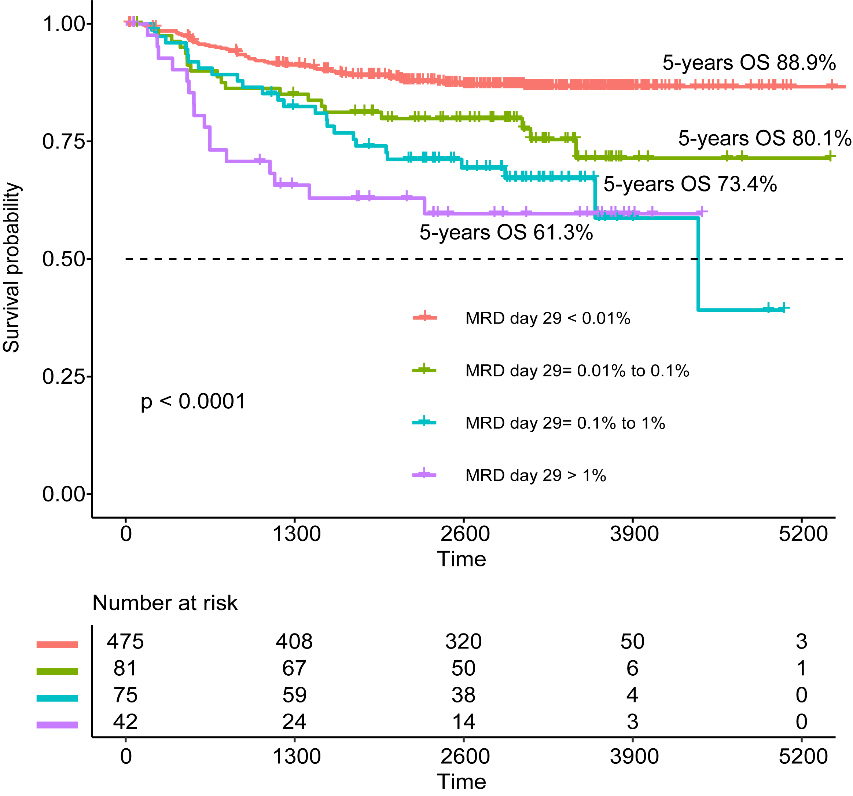


**Supplementary Figure 4.** KM analysis of MRD day 29.
